# Laser Nanostructuring of Titanium Surfaces for Enhanced Bioactive Applications

**DOI:** 10.3390/ma18102362

**Published:** 2025-05-19

**Authors:** Angela De Bonis, Mariangela Curcio, Agostino Galasso, Nicola Caggiano, Antonio Lettino, Patrizia Dolce, Donato Mollica, Maria Lucia Pace, Antonio Santagata

**Affiliations:** 1 Dipartimento di Scienze di Base e Applicate, Università Degli Studi Della Basilicata, Viale Dell’ateneo Lucano 10, 85100 Potenza, Italy; angela.debonis@unibas.it (A.D.B.); mariangela.curcio@unibas.it (M.C.); agostino.galasso@unibas.it (A.G.); 2 Dipartimento di Chimica, Università Degli Studi di Bari, Via Orabona 4, 70125 Bari, Italy; nicola.caggiano@uniba.it; 3 IMAA-CNR, Tito Scalo, Zona Industriale, 85050 Potenza, Italy; antonio.lettino@imaa.cnr.it; 4 ISM-CNR, FemtoLAB, Tito Scalo, Zona Industriale, 85050 Potenza, Italy; patrizia.dolce@ism.cnr.it (P.D.); donato.mollica@ism.cnr.it (D.M.); marialucia.pace@ism.cnr.it (M.L.P.)

**Keywords:** laser nanostructuring, titanium surfaces, LIPSS, bioactive nanostructures, surface functionalization, biomedical applications

## Abstract

Laser nanostructuring via Laser-Induced Periodic Surface Structures (LIPSS), generated using femtosecond laser pulses, has been investigated as a method for precisely modifying titanium surfaces. By adjusting parameters such as the fluence and pulse number of the laser beam, it is feasible to tailor the surface morphology, roughness, and oxidation states of species that can significantly influence the properties and surface bioactivity of the material. In this study, the LIPSS was applied to commercially pure titanium and evaluated for its ability to support calcium phosphate nucleation and growth in Simulated Body Fluid (SBF). Scanning Electron Microscopy (SEM) and Fast Fourier Transform (FFT) analysis confirmed the formation of well-defined periodic structures. Additional characterizations performed by Atomic Force Microscopy (AFM) and X-ray Photoelectron Spectroscopy (XPS) revealed, after laser treatment of titanium, its increased surface roughness and oxidation levels, respectively. These features, when assessed after immersion in SBF, were associated with an improved potential biological performance of the nanostructured surface of the investigated material. The results demonstrated that LIPSS-treated titanium effectively promoted calcium phosphate growth, indicating its enhanced potential bioactivity. Overall, LIPSS nanostructuring presents a scalable and cost-effective strategy for engineering titanium surfaces with potential bioactive properties, supporting their promising application in advanced biomedical implants.

## 1. Introduction

In the vast landscape of scientific research, the exploration of nanostructured materials has become increasingly demanding, driving scientists and engineers to develop innovative and sustainable techniques. Among these, laser nanostructuring has emerged as a promising method for modifying the properties of metals, expanding their applications in both technological and biomedical fields.

A particularly fascinating aspect of this technique is the formation of Laser-Induced Periodic Surface Structures (LIPSS), patterns that arise from the interaction between ultrashort pulsed lasers and the matter. These structures are influenced by multiple factors, including the properties of laser radiation and the physical–chemical features of the material such as its composition. Over time, studies have revealed that the periodicity and orientation of these structures depend strictly on the laser parameters such as the wavelength of the applied laser beam as well as its polarization [1,2,3].

LIPSS can be divided into two main categories based on their periodicity: Low-Spatial Frequency LIPSS (LSFL) and High-Spatial Frequency LIPSS (HSFL). LSFL exhibit periodicities between half and the full wavelength of the laser radiation, while HSFL can reach dimensions as small as a tenth of the laser wavelength. LSFL are particularly common in metallic materials and their paths are strongly dependent on the polarization of the laser beam used [4]. Conversely, HSFL emerge under high-density irradiation conditions, where additional interference effects and plasmonic resonances play a significant role, leading to finer, more complex periodic structures [5].

The formation of LIPSS is dictated by several other laser parameters, including fluence, the number of pulses applied, pulse duration, and the angle of incidence. Controlling these factors enables the creation of tailored surface structures with unique properties, making a LIPSS a versatile technology suitable for numerous applications [6,7,8,9,10,11,12,13,14,15,16,17,18,19,20,21] such as industrial and biomedical ones.

From a theoretical standpoint, the formation of LIPSS is explained through complex models involving electromagnetic interactions and material reorganization mechanisms.

The two-temperature model describes the laser–metal interaction as an energy exchange between electrons and the crystal lattice, linking surface features to pulse duration and material properties. The electromagnetic model explains LIPSS formation through the interaction of incident radiation with surface electromagnetic waves or surface plasmon polaritons [22,23,24]. The Sipe model suggests that surface roughness selectively absorbs radiation, defining the periodicity and orientation of the structures. LIPSS formation may also result from material reorganization driven by hydrodynamic effects and phase transitions caused by temperature and pressure gradients [2,3].

In the biomedical field, titanium is highly valued for its ability to integrate with biological tissues without causing adverse reactions. Its bioinertia and excellent mechanical properties make it a widely used material in prosthetics and orthopedic implants. However, recent research has investigated whether laser nanostructuring can enhance its durability while also inducing bioactivity, which allows the material to promote the growth of biological tissues on its surface [25]. While prior studies have characterized Laser-Induced Periodic Surface Structures (LIPSS) under various laser conditions, the systematic exploitation of these patterns to induce targeted bioactivity on titanium implants still needs to be investigated.

Here, a comprehensive methodology employing a femtosecond Ti-Sapphire laser to generate and tailor the LIPSS on titanium, coupling precise laser parameter optimization with in-depth bioactivity assessment, is introduced. By varying key processing parameters such as the fluence, pulse number, and hatch interline spacing, reproducible links are established between the processing conditions and resulting nanostructure morphology and chemistry.

To analyze these modified surfaces, advanced characterization methods such as X-ray Photoelectron Spectroscopy (XPS), Scanning Electron Microscopy (SEM), and Atomic Force Microscopy (AFM) were employed, providing insight into how femtosecond pulses reorganize titanium’s surface at both the atomic and nanoscale levels.

The LIPSS experiments performed using titanium samples have been conducted to explore the induced bioactivity, dipping the obtained nanostructured surfaces in a Simulated Body Fluid (SBF) solution to observe the nucleation of apatite, highlighting the enhanced osseointegration potential, correlated to the tailored LIPPS geometry [26].

## 2. Experimental and Methods

### 2.1. Preparation of Nanostructured Titanium Surfaces

The titanium samples were initially pre-treated using sandblast paper of different grit sizes to make the surface as homogeneous as possible. Subsequently, they were immersed for 60 s in an aqueous solution composed of 5% of sulfuric acid and 30% nitric acid to remove the TiO_2_ layer. After this step, the samples were thoroughly washed with distilled water to remove any chemical residues and prevent contamination, ensuring that any modifications to the surface chemistry could be attributed exclusively to the laser treatment.

To optimize the working conditions, a titanium sample measuring 4.0 cm × 4.0 cm per side was used, on which a pattern of several nanostructured areas of 0.2 cm per side were generated to relate the laser parameters employed with the nanostructures obtained, with the approach following the methodology used in a previous study [18]. For bioactivity evaluation, samples measuring 1.0 cm × 1.0 cm with a nanostructured area of 0.8 cm per side were used. The nanostructuring and production of LIPSS on the titanium samples were performed using a Spitfire Pro XP Ti-Sapphire laser (Spectra Physics, Milpitas, CA, USA), characterized by ultrashort pulses with a duration of 120 femtoseconds, operating at a wavelength of 800 nm and with a repetition rate of 1 kHz.

The scanning process was carried out using a Newport Corporation µFAB workstation (Irvine, CA, USA); the system is equipped with a set of microscope objectives, capable of focusing the laser beam onto the sample with diverse spots, and a software-controlled attenuator for adjusting the laser energy impinging onto the sample surface. The µFAB workstation also features a remote-controlled x,y,z mobile platform on which the sample was positioned, allowing the selection in μm/s of the sample holder scan speed and, consequently, the dose of laser beams interacting with the exposed areas of the moving sample. The Newport µFAB workstation was managed through the manufacturer 3.9.10 dedicated software, which enabled the designed hatch tracing to achieve a uniform LIPSS nanostructured area. In all experiments performed in this study, the laser was focused using a microscope objective (Olympus Plan N 4× objective, Olympus, Tokyo, Japan) with a numerical aperture (NA) of 0.01 and a focal length of 18.5 mm. The final focused laser spot diameter was 20 µm.

To ensure maximum control over the process, some key parameters were adjusted such as the following: (1) the number of pulses per unit area (N per µm⁻^2^), which was modified by adjusting the laser beam scanning speed between 1000 µm/s and 5000 µm/s; (2) the fluence (Φ), determined by the energy of the incident radiation per surface area unit, expressed in J/cm^2^, and varied between 0.14 J/cm^2^ and 0.28 J/cm^2^ through the percentage of the energy of the incident laser beam that was effectively transmitted, selected at the attenuator; and (3) the hatch interline spacing, defined as the distance between two successive scans, with tested values of 50 µm and 25 µm.

### 2.2. Bioactivity Test (Kokubo Method)

The assessment of the sample bioactivity was conducted using the Kokubo test [26]. The nanostructured titanium samples under the optimal conditions selected in the initial phase of the study were placed in vials containing an amount of SBF solution, expressed in ml, which was quantified by the apparent surface area of the sample “Sa” used through the formula V = Sa/10, where “Sa” represents the apparent surface area of the sample expressed in mm^2^, which in this case was 64 mm^2^. The vials were placed in a bath at 37.0 °C, and the SBF solution was replaced with a new one every 3 days to maintain the solution’s ionic concentration during the incubation time. The stability of the stock solution was monitored through pH measurements.

The samples were analyzed after being in contact with the solution for periods of 14 and 28 days.

### 2.3. Atomic Force Microscopy (AFM)

AFM analyses of the titanium samples were performed in contact mode using a PARK XE-120 Atomic Force Microscope (Park Systems, Burlington, MA, USA) and processed using XEI 4.3 software. Roughness was calculated as the surface area ratio (*SAR*):

(1) $$SAR\;\left(\%\right)=\frac{{Area}_{eff}-{Area}_{geom}}{{Area}_{geom}}\;\cdot 100$$

This value represents the percentage increase in the area effectively scanned by the instrument’s tip compared to the relative geometric area. Four different magnifications were acquired: 30 µm × 30 µm, 20 µm × 20 µm, 10 µm × 10 µm, and 5 µm × 5 µm. Additionally, during data processing, the R_MAX_ value was extracted, considering the maximum height difference between the peak and valley of the laser-induced nanostructures. This value was averaged over three measurements for each image to ensure a minimum set of suitable data.

### 2.4. X-Ray Photoelectron Spectroscopy (XPS)

The chemical surface characterization of the samples was performed using X-ray Photoelectron Spectroscopy (XPS) with the PHOIBOS 100-MCDS spectrophotometer (SPECS, Berlin, Germany). The source and sample were placed in an ultra-high-vacuum chamber with a pressure of 10⁻^9^ mbar. The source used was an aluminum anode. The detection limits in atomic percentage ranged from 0.1% to 1.0%. Spectra were acquired using the Fixed Analyzer Transmission (FAT) configuration with an analyzer pass energy of 9.0 eV. The resolution for the wide spectrum was 10.0 eV, while for detailed element analyses, it was 1.0 eV. The signal fitting was performed using the customized Newgoogly software, and the signals were compared with the literature data and the NIST database.

### 2.5. Scanning Electron Microscopy and Energy Dispersive X-Ray Spectroscopy (SEM–EDS)

SEM–EDS analyses were carried out using a Zeiss Supra 40 Scanning Electron Microscope (Zeiss, Oberkochen, Germany) coupled with an Oxford Inca Energy 350 X-ray Dispersive Spectrometer (Oxford Instruments, Abingdon, UK). The use of a Field Emission source allowed high-resolution imaging, whereas the EDS system could detect elements with *Z* > 4 with a quantitative analysis uncertainty of 1–2%. Images were acquired at different magnifications using a voltage of 15 kV and an aperture of 6 mm. For titanium samples not dipped into the SBF solution, no metallization was required; conversely, the other samples were coated with a thin graphite film layer. The periodicity (Λ) of the nanostructures was determined using Fourier Transform (FT) performed with ImageJ processing 1.54g software; each image of every sample acquired was divided into four quadrants, with FT applied to each of these, and their values were averaged.

## 3. Results and Discussion

### 3.1. Effect of Experimental Parameters on the LIPSS

The study of the effects induced by the experimental conditions used was conducted by keeping two of the three analyzed variables constant for each observed aspect. Initially, for the study of the number of pulses per unit area (N) and fluence (Φ), the hatch spacing was fixed at 50 µm.

Below, a representative selection of SEM images are presented, acquired at 30,000× magnification for titanium surfaces structured at different experimental conditions. To evaluate the ripples’ distance, the image profile was considered and the distances of more than 20 peaks were evaluated.

**Figure 1:**
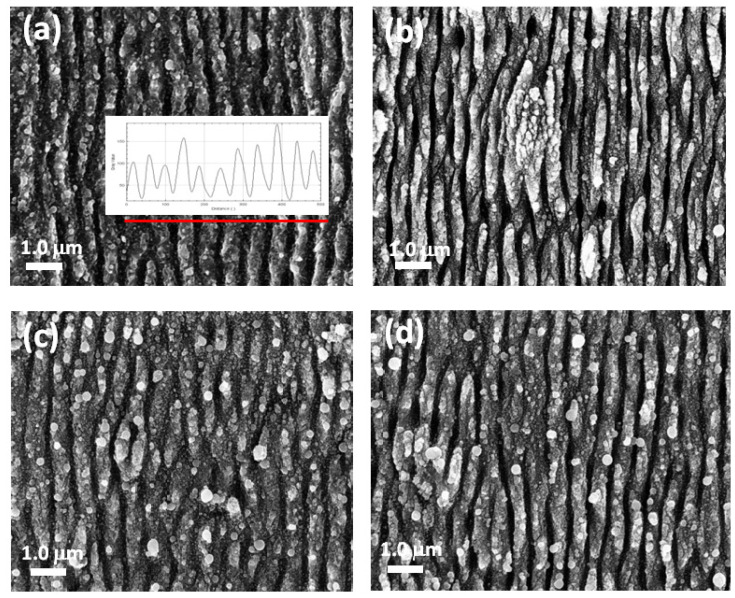
SEM images of nanostructures obtained at (**a**) Φ = 0,19 J/cm^2^, N = 25 µm^−2^. In the inset, the plot profile of the ripples crossed by the red line is reported; (**b**) Φ = 0,19 J/cm^2^, N = 75 µm^−2^; (**c**) Φ = 0,14 J/cm^2^, N = 38 µm^−2^; (**d**) Φ = 0,28 J/cm^2^, N = 38 µm^−2^. The dimension bar is of 1 μm.

The samples shown in Figure 1a,b were prepared at the same fluence and with an increasing N value. The distance of the ripples (periodicity Λ) was in the range of 580 ± 20 nm and 440 ± 20 nm.

Subsequently, the focus shifted to fluence variation. The reported SEM images (Figure 1c,d) show the nanostructure patterns obtained by increasing the fluence and keeping N = 19 µm⁻^2^. A direct proportionality between periodicity and fluence was observed, with values of 520 ± 10 nm for Φ = 0.14 J/cm^2^ and 580 ± 10 nm for Φ = 0.28 J/cm^2^. However, at a higher fluence, the sample appeared inhomogeneous, showing a high presence of droplets and fusions in the periodic pattern. Since the goal was to achieve a homogeneous distribution of nanostructures, it was advisable to consider lower fluence values instead.

**Figure 2:**
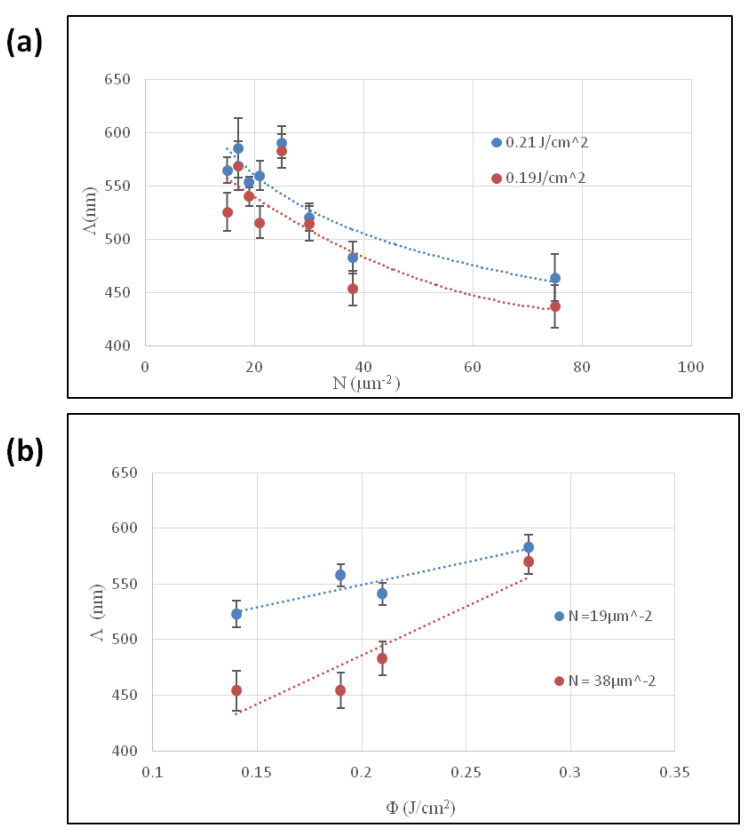
The effect of the laser parameters on the Λ: (**a**) effect of number of pulses per unit area (N); (**b**) effect of fluence (Φ).

In Figure 2, graphs are presented, displaying how the periodicity was affected by using different parameters. At the fluencies considered, by increasing the N value, a decreasing distance between the ripples was observed, ranging from Λ above 550 nm for N = 15 to a value below 450 nm for N = 78, for a fluence of 0.19 J/cm^2^ where the length of the regular linear ripples was affected. On the other hand, increasing the fluence led to an increase in the periodicity, regardless of the N values.

To ensure the applicability of the process on a larger scale, it is essential to maintain sample homogeneity. Reducing the hatch distance significantly decreases the non-processed areas, making the sample surface more homogeneous. This strategy can improve nanostructure density which could also be beneficial in increasing sample roughness and, consequently, its potential bioactivity.

### 3.2. Topographic and Compositional Analysis

Because surface roughness can be a determining factor for establishing the bioactivity of these materials, the laser-induced nanostructures have been investigated by AFM. Since it has been observed that higher fluencies can induce inhomogeneities on the nanostructured surfaces, a fixed fluence of 0.19 J/cm^2^ and different values of N and hatch distance have been considered. The experimental conditions used to prepare the samples analyzed by AFM are reported in the following table.

**Figure 3:**
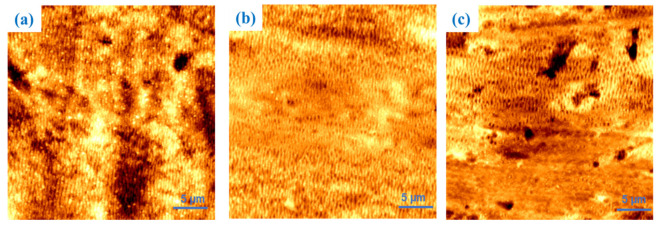
AFM images of samples 1 (**a**), 2 (**b**), and 3 (**c**). An area of 30 µm × 30 µm was scanned for each image.

Figure 3 presents 30 µm × 30 µm AFM scans of these samples, identified according to the numbering reported in Table 1.

Samples 1 and 2 were produced with a hatch of 25 µm, while sample 3 was generated with a hatch of 50 µm. Sample 1 exhibits periodicities of 510 ± 20 nm, sample 2 of 550 ± 15 nm, and sample 3 of 500 ± 20 nm.

Samples 1 and 2 exhibit a high degree of nanostructure distribution homogeneity, whereas sample 3, due to its larger hatch value, shows areas with various degrees of processing. Table 2 summarizes the measured surface area ratio values, which are lower for sample 3 (i.e., 10.4%) than the others. This discrepancy may be related to the presence of unprocessed regions, as can be observed in the lower area of Figure 3c.

**Figure 4:**
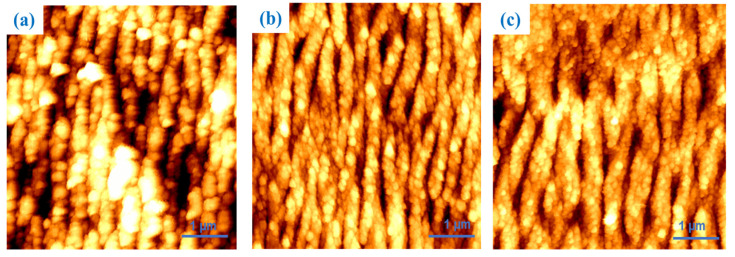
AFM images of samples 1 (**a**), 2 (**b**), and 3 (**c**). An area of 5 µm × 5 µm was scanned for each image.

At a higher magnification (Figure 4), all samples exhibit a higher roughness, suggesting the concept that the roughness is nanometric in nature. Moreover, sample 2 presents a higher value compared to the others, which is assumed to be due to its higher periodicity. Additionally, the R_MAX_ for samples 1, 2, and 3 were 145 ± 50 nm, 160 ± 50 nm, and 150 ± 35 nm, respectively. Since these values do not significantly differ from each other, the greater roughness is attributed to the different LIPSS periodicities rather than their variations in depth.

Based on the characterization data for nanostructure-controlling parameters and the subsequent topographical analysis, the optimal nanostructuring conditions were determined to be Φ = 0.19 J/cm^2^, l = 25 µm, and N = 19 µm⁻^2^. In fact, sample 1 was excluded from the nanostructuring condition since, despite having nanostructure homogeneity like sample 2, its roughness was not comparable to this. It is likely to be displayed in sample 1 because of the nanostructure modification induced by exceeding the dose of the impinging laser beam; when compared to sample 2, it can be seen that it negatively affects this parameter. Sample 3 was also excluded as it failed to meet both criteria.

Since surface chemistry is key determinant parameter for assessing a material’s potential bioactivity, alongside roughness [27], a survey has been provided on the generated nanostructures relating to how surface composition can be altered by LIPSS and, consequently, how bioactivity could be affected by it. The analyzed samples reported here correspond to those with roughness measurements listed in Table 2 and include a reference titanium sample, so that a comparative analysis of LIPSS with different periodicities could be displayed.

**Figure 5:**
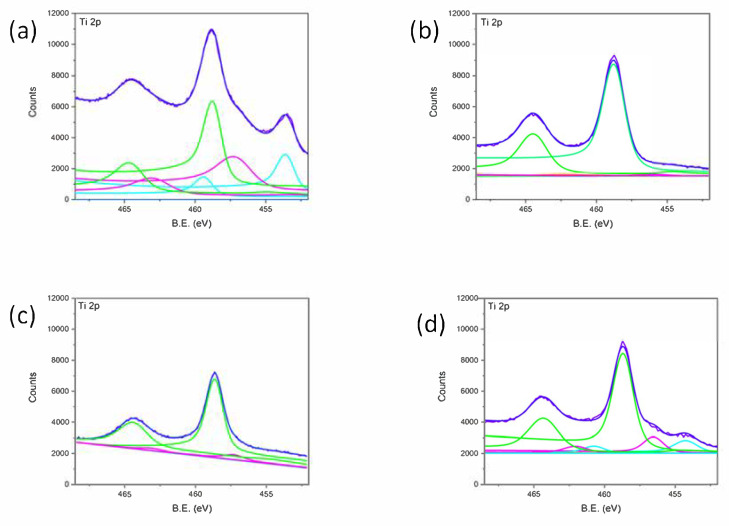
Ti 2p region for (**a**) bare titanium before its laser treatment, whereas (**b**–**d**) are related to the nanostructured samples 1, 2, and 3, as reported in Table 2.

The Ti 2p spectra within the 452–467 eV range are presented in Figure 5 for all of the analyzed samples. The spectrum of Figure 5a can be resolved in four contributions, assigned respectively to metallic Ti (Ti 2p_3/2_ at 454.3 eV), TiO (Ti 2p_3/2_ at 455.0 eV), Ti_2_O_3_ (Ti 2p_3/2_ at 456.8 eV), and TiO_2_ (Ti 2p_3/2_ at 458.8 eV).

Considering samples 1, 2, and 3, the findings are aligned with the existing literature, confirming that laser-treated titanium exposed to air results in an oxide layer formation [2,7,12]. The outermost layer consists of stoichiometric Ti (IV) oxide, while sub-stoichiometric oxides are found underneath. As is shown in Table 3, nanostructuring leads to increased oxidation, evidenced by the absence or residual amount of metallic Ti and sub-stoichiometric TiO signals. Among the samples, sample 3 exhibited the lowest oxidation level. The processing inhomogeneity is evident from the Ti (0) signal, which is less prominent or absent in samples 1 and 2.

In summary, nanostructuring induces changeable degrees of oxidation in all samples, depending on the nanostructure texturing. The literature findings indicate that the natural oxide layer on titanium enhances its biocompatibility by preventing undesirable chemical reactions [25,28]. Furthermore, Ti (IV) oxide in a mildly basic solution can present Ti-O⁻ groups, facilitating the nucleation of calcium phosphate by initially coordinating Ca^2^⁺ ions, followed by phosphate ions [26].

Based on these findings, the optimal nanostructuring parameters for promoting bioactivity, due to the combination of roughness and the oxidation degree, correspond to sample 2, with Φ = 0.19 J/cm^2^, I = 25 µm, and N = 19 µm⁻^2^. This sample exhibits Λ comparable to the literature-reported values that are proven effective for biocompatibility [25,28], along with high roughness and a well-balanced oxidation level between samples 1 and 3.

### 3.3. In Vitro Bioactivity Assessment

**Figure 6:**
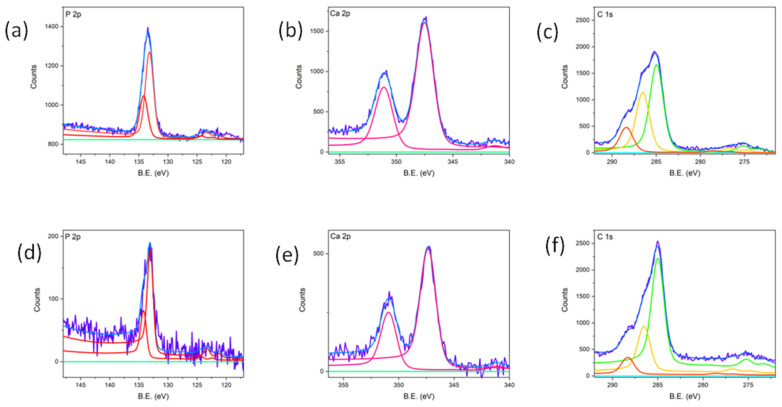
XPS analysis of sample 2 after 14 (upper line) and 28 days (lower line) of soaking in SBF. (**a**,**d**) P2p; (**b**,**e**) Ca2p; (**c**,**f**) C1s.

As is shown here, the present study is focused on assessing the potential bioactivity induced by the nanostructuring of titanium samples under the previously selected LIPSS processing conditions. The nanostructured titanium surfaces were soaked in SBF solutions and analyzed at 14 and 28 days. XPS analysis for the sample soaked for 14 days in SBF confirmed the formation of a calcium phosphate phase. Figure 6 presents the detailed outcoming spectra related to Ca 2p, P 2p, and C 1s signals. The P 2p spectrum highlights the presence of phosphates on the sample surface, identified by the characteristic binding energy (BE) signal at 133.0 eV. Regarding Ca, the peak position of 2p_3/2_ at 347.4 eV confirms the presence of calcium phosphate. However, this BE range also includes the signal of calcium carbonate [29]. The detailed C 1s spectrum shows three contributions at 285 eV, 286.3 eV, and 288.3 eV, assigned to aliphatic carbon, C-O, and C=O species, respectively. No contribution is present at 289.0 eV, indicating the absence of carbonate-like species and suggesting that that Ca is entirely bound to phosphate.

Through quantitative analysis, the Ca/P ratio for this sample was 0.92. This finding is significant when compared to the literature data, which indicate that non-nanostructured Ti samples tested under the same SBF conditions do not exhibit any calcium phosphate growth [26].

To further investigate the effect of prolonged exposure, a subsequent sample was immersed in the SBF solution for 28 days, and its detailed spectra were generated. The P 2p spectrum confirms the presence of phosphorus in the form of phosphate, identified by the signal at 133.0 eV. Similarly, Ca was exclusively present as calcium phosphate, as evidenced by the absence of carbonate-related signals in the C 1s spectrum.

The calculated Ca/P ratio from quantitative analysis was 1.10. The presence of calcium phosphate on the surface is indicative of the material’s potential bioactivity, making its growth a crucial factor. Additionally, calcium phosphate can exist in biological systems in non-stoichiometric forms, which may serve as an intermediate phase towards the formation of a more stable phosphate structure [26].

SEM–EDS analysis of nanostructured sample after 14 and 28 days of soaking in SBF was conducted to examine surface morphology.

**Figure 7:**
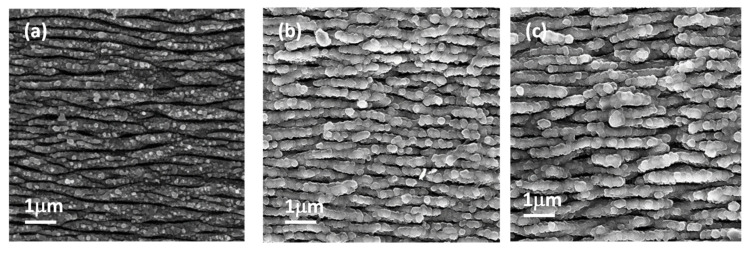
SEM images: (**a**) nanostructured titanium; (**b**) nanostructured titanium after soaking in SBF for 14 days; (**c**) nanostructured titanium after soaking in SBF for 28 days.

The images (Figure 7b,c) show the formation of globular structures that increase with the soaking time and that cover the titanium nanostructures while preserving the interstitial spaces between them. The cauliflower-like morphology of the structures grown during the SBF test is characteristic of apatite-like formations [30].

The EDS analysis also confirmed the presence of Ca and P, with a calculated ratio of 1.33 ± 0.34, and the value was comparable to the Ca/P ratio evaluated by XPS analysis.

## 4. Conclusions

LIPSS nanostructuring represents a practical and cost-effective approach to modifying the physicochemical properties of a material. The use of a femtosecond laser opens interesting possibilities in this context, particularly the ability to generate sub-wavelength periodic structures with characteristics and properties that change through the variation in the operating conditions.

The morphological features of the produced structures, performed using scanning electron microscopy (SEM) and Fourier Transform (FT) analysis of the acquired images, indicated that the periodicity tends to decrease with an increasing number of pulses per unit area (N), whereas by increasing the laser fluence, the periodicity is affected in the opposite way.

For practical technological applications, LIPSS production must be uniform across the entire sample, avoiding phenomena such as melting or redeposition. Consequently, the parameter selection focused on samples produced with a low fluence value (0.19 J/cm^2^) and a scan hatch spacing of 25 µm was employed.

Additionally, surface roughness was evaluated through atomic force microscopy (AFM), which related surface roughness to the periodicity of the formed nanostructures rather than the depth of the grooves induced during laser processing. Samples 1 and 2 showed the highest roughness value at different magnifications, revealing both micrometric and nanometric roughness.

The sample prepared at ϕ = 0.19 J/cm^2^, N = 19 µm⁻^2^, and a hatch of 25 µm (sample 2) exhibited increased surface oxidation and a higher roughness degree and was considered for the in vitro bioactivity test.

The results demonstrated that nanostructured titanium exhibits the ability to induce apatite formation on its surface, confirming its potential bioactive features and making them promising candidates for biomedical applications. Furthermore, the bioactivity of these titanium surfaces could be further enhanced by the deposition of thin bioactive coatings, such as bioglass or ceramic films. These coatings may not only improve the material’s ability to promote bone-like apatite formation but also enhance other critical properties, such as corrosion resistance, mechanical stability, and long-term biocompatibility. Future studies should focus on optimizing the combination of laser nanostructuring and bioactive coatings to develop advanced titanium-based materials with superior performance for clinical applications.

## Figures and Tables

**Table 1:** Experimental condition for the laser-induced nanostructures investigated by AFM.

Sample	Φ (J/cm^2^)	l (μm)	N(μm^−2^)
1	0.19	25	75
2	0.19	25	19
3	0.19	50	15

**Table 2:** SAR (%) evaluated for 30 × 30 μm and 5 × 5 μm AFM images.

Sample	30 × 30 μm	5 × 5 μm
1	22.1	47.9
2	25.5	76.0
3	10.4	47.9

**Table 3:** Titanium chemical composition evaluated by XPS measurements.

%	Before LIPPS	Sample 1	Sample 2	Sample 3
Ti	19	-	6	11
TiO	5	-	-	-
Ti_2_O_3_	18	2	10	12
TiO_2_	58	98	84	77

## Data Availability

The original contributions presented in this study are included in the article.
